# Healthcare Social Workers’ Scope of Practice during COVID-19

**DOI:** 10.3390/healthcare12020174

**Published:** 2024-01-11

**Authors:** Tiffany Washington, Terri D. Lewinson

**Affiliations:** 1 School of Social Work, University of Georgia, Athens, GA 30602, USA; 2 The Dartmouth Institute for Health Policy and Clinical Practice, Dartmouth College, Hanover, NH 03755, USA; terri.d.lewinson@dartmouth.edu

**Keywords:** healthcare, COVID, social work, scope of practice

## Abstract

The COVID-19 pandemic pushed the U.S. healthcare system to its limits, resulting in the need for flexibility in care delivery. This study aimed to describe healthcare social workers’ scope of practice since the start of the pandemic. Semi-structured interviews for this qualitative study were conducted using the Zoom platform between July and August 2020. This study used a basic qualitative content analysis with integrated deductive and inductive coding to explore participant perspectives. Their scope was assessed based on healthcare social work practice standards. Four practice standards and eight themes that emerged from the data were knowledge and skills (care planning and intervention and social worker–patient relationship), workload sustainability (workload expansion and workload facilitators), interdisciplinary collaboration (collaborating beyond the scope of responsibilities and collaboration challenges), and cultural competency (institutional and societal). The findings add a deeper understanding of the roles social workers perform, how they think about these roles, how they want to be understood, and how they are best utilized in ways consistent with their training and expertise. Moving forward, healthcare systems may consider well-delineated roles and responsibilities for everyday practice and during pandemics.

## 1. Introduction

The COVID-19 pandemic pushed the U.S. healthcare system to its limits, resulting in the need for flexibility in care delivery. The Centers for Medicare & Medicaid Services (CMS) and state government officials adjusted legislation that impacted healthcare professionals’ scopes of practice. For example, CMS removed nurse practitioners’ practice restrictions requiring physician supervision, allowed hospice home health aides to practice past their required annual supervisory visits [1], and allowed dialysis care teams to delay new patients’ care plans beyond 30 days [2]. In addition, reliance on telehealth increased, the healthcare workforce declined [3], and medical providers practiced across state lines [4].

Fortunately, social workers in clinical practice were allowed to provide teletherapy for Medicare beneficiaries [5]. Beyond teletherapy, little is known about how social work practice has impacted hospitals, dialysis facilities, skilled nursing care, and other healthcare settings. A limited understanding of social workers’ scope of practice existed before the pandemic. It is unclear how their practice scope has been impacted since the pandemic. This study aims to address this literature gap because, in healthcare settings, a lack of clarity on social workers’ roles relates to unpleasant synergic team experiences, patient outcomes, care, and interdisciplinary collaboration [6,7,8,9].

## 2. Background

### 2.1. Healthcare Social Work

Over one-quarter of the social work workforce is in healthcare settings [10]. Healthcare social work (HSW) is one of the fastest-growing occupations, with a projected annual need of 747,000 workers [10]. They provide psychosocial support to individuals and families affected by chronic conditions. Because of healthcare’s fast pace, HSWs perform rapid assessments to identify strengths and unmet needs (e.g., social support and depression) and implement brief interventions to improve patient wellbeing [11]. HSWs become experts in their practice area and are typically tasked with providing health education [12]. For instance, oncology social workers are knowledgeable about types of cancer, treatment options, and cancer-specific community resources. Likewise, nephrology social workers are knowledgeable about the kidney transplant process, transplant disparities, and requirements for transplant eligibility.

Primarily, HSWs conduct psychosocial assessments on patients’ presenting family history, cognitive and mental health status, financial needs, emotional health, and other aspects of psychological functioning and social environment [13]. Secondarily, HSWs provide case management based on unmet needs identified in the psychosocial assessment. Case management involves referrals to community resources, outpatient providers, and social service workers. Finally, depending on the setting, HSWs contribute to discharge or ongoing care planning to improve health [14]. The quality and quantity of social work’s contribution in these roles is dictated by CMS, making social work integral and necessary in healthcare settings [12].

### 2.2. Social Work and COVID-19

Despite social work’s increased presence in healthcare before COVID-19, the profession’s role was woefully misunderstood by healthcare colleagues [7]. In fact, previous studies found this ambiguity to contribute to inappropriate referrals, exclusion from health policy reform, overlapping and poorly defined roles, and uncertainty about social work’s involvement in patient care [7,15,16,17]. This lack of understanding of HSWs’ significance and confusion and misinformation about transmission, safety, and infection control at the start of the pandemic [18] led to concerns about how social work would be utilized during COVID-19.

At the same time, the pandemic is an opportunity to educate the medical community about HSWs’ roles and training. First, the pandemic increased awareness about social determinants of health, racism in healthcare, and health inequities [19,20], thereby also creating an opportunity to educate the medical community about HSWs’ roles and training. Social workers have a long tradition of identifying and addressing social determinants of health (SDOH) through assessment and intervention, with social justice as the profession’s unifying core value and the lens through which they practice [21,22]. Furthermore, the COVID-19 pandemic activated social workers as intermediaries between patients and families who could not access them [23]. They, too, played a supportive role in addressing the socio-emotional needs of their interdisciplinary team members. For these reasons, the value of social work practice in healthcare settings is worth highlighting.

### 2.3. Scope of Practice Defined

As a conceptual framework, this study used the American Medical Association’s (AMA) definition of the scope of practice for healthcare professionals and the National Association of Social Workers’ (NASW) Standards for Social Work Practice in Health Care Settings. AMA defines scope of practice as “the activities that a person licensed to practice as a health professional is permitted to perform” [24]. Their activities are restricted to laws and statutes within their state of practice. Further, NASW offers 13 practice standards in healthcare settings [25]. The standards delineate the required competencies for ethical and quality social work practice in healthcare environments.

### 2.4. Study Purpose

The objective of this study was to describe HSWs’ scope of practice since the start of the pandemic. HSWs’ roles are not well understood, and little is known about how those roles evolved during COVID-19. The research question was, “What is the impact of the coronavirus crisis on social workers’ scope of practice?” Data were extracted from the Healthcare Social Workers’ Scope of Practice during COVID-19 qualitative dataset.

## 3. Method

### 3.1. Participants

Purposive sampling methods were used to recruit HSWs through email announcements, social media posts, and snowball sampling. The recruitment flyer listed an email address for potential participants to inquire. They received a Qualtrics survey link to answer the prescreening questions and consent procedures. Eligible participants were social workers by degree (i.e., BSW or MSW) and worked at least 30 h per week in a healthcare setting for six months or longer. Participants meeting the inclusion criteria received an auto-generated email to the participant with a confirmation of their interview time and a private Zoom link.

### 3.2. Interview

Semi-structured interviews for this qualitative study were conducted using the Zoom platform between July and August 2020. Interviews were recorded and imported into Otter.ai for transcription. Background information was obtained and entered into Excel. This Excel sheet was stored in a password-protected university-shared Onedrive, only accessible to the Co-Principal Investigators (co-PIs). Three graduate research assistants independently edited transcriptions for clarity. Transcribed interviews were then imported into a password-protected university shared drive only accessible to study team members. The co-PIs conducted the interviews at 40 to 60 min in duration.

The lead author developed the initial interview guide. The co-PIs met twice to refine the questions and ensure they related to the overarching research questions. The co-PIs met after completing three interviews each to refine the questions. Similar to previous qualitative studies examining medical professionals’ scope of practice, the questions were based on characteristics of their roles described in the literature [26] and broadly focused on skills, knowledge, and interactions [27]. An example item was, “What is the scope of your work in your healthcare setting?” Also, trust and rapport were established because of the co-PIs’ extensive healthcare social work practice backgrounds [28]. Participants were assigned pseudonyms for confidentiality. Participants were emailed USD 20 electronic Amazon gift cards after completing their interviews. This study was approved by the University of Georgia Institutional Review Board. The interview guide is available in the Appendix A.

### 3.3. Data Analysis

This study used a basic qualitative content analysis with integrated deductive and inductive coding to explore participant perspectives of MSW practice during COVID-19. This approach allowed for categorizing the data based on the existing scope of practice frameworks to guide the inductive open coding [29]. First, the research team repeatedly listened to audio-recorded interviews while reading transcripts to become familiar with the data [29]. Because this study aimed to understand HSWs’ practice activities during the pandemic, NVivo (v. 14.23) was used to deductively condense transcript text into 13 manageable a priori code categories consistent with NASW’s well-defined 13 practice standards in healthcare settings. According to these standards, social work practice must be grounded in activities that consider (1) ethics and values, (2) qualifications, (3) knowledge, (4) cultural and linguistic competence, (5) screening and assessment, (6) care planning and intervention, (7) advocacy, (8) interdisciplinary and inter-organizational collaboration, (9) practice evaluation and quality improvement, (10) record-keeping and confidentiality, (11) workload sustainability, (12) professional development, and (13) supervision and leadership. Next, four coders inductively open-coded short phrases representing distinct practice activities within each code category and applied in-vivo or researcher-derived labels to these data bits. Clustered data bits were repeatedly expanded, collapsed, and reorganized to compare, contrast, and identify patterns. To reach a consensus, the coding team shared analytic memos intended to interpret the data patterns and meaning. Category labels and content were iteratively adjusted to include new text as needed. New categories were formed for text with practice-relevant constructs that did not conceptually fit in the original a priori titles. Categories that did not include any relevant text from this study sample were dropped. This iterative process continued until four of the 13 NASW categories were retained: knowledge and skills, workload sustainability, interdisciplinary collaboration, and cultural collaboration. A summative description of participants, categories, and themes are described below and displayed in Table 1 and Table 2, respectively.

Four of the 13 healthcare social work practice standards were most prevalent in the data. Those were knowledge and skills, workload sustainability, cultural competency, and interdisciplinary collaboration. To maintain anonymity, direct quotes are not associated with interviewee demographics.

## 4. Findings

### 4.1. Participants

In this sample of 50 women and four men social workers (N = 54), most participants were White (59.2%) and between ages 23 and 30 (48.1). These social workers were primarily masters-trained (98.1%) and licensed (85.2% were LMSW or LCSW). Regionally, most social workers were in the southern United States (57.4%), in hospitals (63.0%) and outpatient clinics (24.1). Participants had worked in their settings from 9 months to 19 years.

### 4.2. Scope of Practice: Knowledge and Skills

Social workers require specific knowledge and skills to practice in healthcare systems effectively. These knowledge and skills relate to roles, responsibilities, and client/family engagement in social work intervention.

***Care Planning and Intervention.*** Care planning involves assessing a patient’s presenting health challenges, problem-solving capacities, and identified goals before electing particular social work interventions to help achieve those goals. Participants identified case management as their primary function in the care planning process. Case management involved linking to community resources, coordinating transportation, providing food assistance, ordering durable medical equipment, and managing individualized service plans. Other tasks described were performing mental health screenings and psychosocial assessments, providing brief counseling, facilitating support groups, educating about advanced directives, and helping families navigate end-of-life challenges. Discharge planning was a common task that included, as one emergency room social worker noted, “triaging patients for urgent mental health needs [and] community needs following discharge from the ER.” Over half of the participants regularly engaged in interdisciplinary morning huddles and rounds to discuss patients’ needs, status updates, and care plans.

Interestingly, participants explained that the pandemic increased the severity of patients’ needs. For instance, a hospital social worker said her case management shifted from referrals for food, housing, and education to providing utility assistance and noted, “That’s something we’ve never done before.” Also, an oncology social worker who primarily provided therapy had shifted to helping with “logistical and practical stuff like helping people sign up for assistance” outside of her clinical responsibilities. In addition, social workers stressed that, despite safety policies that prevented families from seeing their loved ones, involving patients’ families and caregivers remained important. “A big part is not only seeing the patients, but the caregivers because … they are facing just as much stress as the patient.” Others described how fairly straightforward pre-COVID-19 case management assistance became more complicated after the pandemic. For instance, a long-term care social worker described how previously helping a resident secure Social Security benefits could take “anywhere from 30 min to 45 min. But now, since COVID-19, an appointment is easily an hour and a half, two hours.” As a result, nearly all participants emphasized the need to involve family caregivers in the care planning process. For instance, one hospital social worker stressed that treating the whole person also meant treating the families and caregivers.

***Social Worker–Patient Relationship.*** Social workers establish relationships with patients for effective care planning. Unfortunately, participants reported limited opportunities to build rapport with patients. For instance, a social worker in an outpatient setting described no longer being able to “speak with patients and family members” during patients’ treatments and said it was “sad” she was unable to provide in-person follow-up. Another outpatient social worker described how all therapy sessions were conducted by phone and distracted by background noise at home. “It’s hard to establish rapport with a patient by phone call…I’m just calling out of the blue,” and he later described how patients are reluctant to “open up” by phone. The lack of face-to-face interaction was repeated in nearly all interviews. One emergency room social worker expressed how the isolation brought patients in because they “don’t know what to do at home.” She stated, “They’re kind of crashing because they’re at home alone. And even though they don’t really want to come to the ER, they’re showing up. They’re coming in because they don’t know what to do at home.” Participants also described how wearing “full protective gear…mask, goggles, face shields, gloves” diminished rapport when they resumed in-person interactions. Another social worker said he can no longer facilitate support groups and that he feels “disconnected” because they were not being offered in person or virtually. Social workers felt concerned for patients who did not have access to at-home technology to maintain contact, with one person calling it an “injustice.”

### 4.3. Scope of Practice: Workload Sustainability

HSWs are required to advocate for workloads that do not interfere with efficient and quality care. Depending on the setting, an excessive workload is associated with emotional exhaustion [30].

***Workload Expansion.*** Nearly all participants described how the COVID-19 pandemic expanded their workload. First, participants experienced an increased patient census and changes to their departmental assignments. For example, one participant described how shifting from outpatient to inpatient care left patients’ needs unmet. Another social worker who covered two outpatient clinics before COVID-19 reduced to one clinic for safety reasons. In addition, some expressed workload changes due to increased remote work. For example, one hospital-based social worker described how working from home every other week has increased his workload. He stated, “On some days, even though I work from home, I’m like I just rather just have an off day because it’s just like, non-stop. As soon as the phone goes [off], sometimes you’re just moving, moving. Sometimes I forget to have lunch.” Interestingly, social workers with clinical tasks described having to take on more case management responsibilities that were solely the role of case managers. One person noted, “We’re having to take on more of contacting EMS, arranging transportation back to facilities, which is stuff that we normally don’t do…contacting physical therapy for consults, were not used to doing that.”

***Workload Facilitators.*** To perform their roles effectively, HSWs described an increased reliance on technology (e.g., telephone and virtual platforms) and providing their personal resources to safely and effectively perform their duties. A hospital social worker explained that he purchased a shredder for home because he had to print documents listing patients’ protected health information. He went on to say that there were discrepancies in resources given to social workers and resources given to other treatment team members. He stated, “But you know, other people that I know that are working from home have been given allowances in terms of buying] stuff for like an office. All I got was my computer.”

Social workers also noted that performing rounds and huddles were interrupted. Some mentioned they were stopped for a time, while others noted they relied on technology to keep them going (e.g., Zoom, telephone, and Facetime). One hospital social worker noted, “We’re not able to fit rounds into the day” due to the limited face-to-face interactions with patients and other team members.

### 4.4. Scope of Practice: Interdisciplinary Collaboration

Team-based collaborations are essential in healthcare settings. HSWs are required to promote interdisciplinary collaborations that enhance and facilitate care delivery.

***Collaborating Beyond Scope of Responsibilities.*** Some HSWs were asked to take a collaborative approach to patient care. In some cases, these requests resulted in HSWs performing outside their practice scope. While some were not asked to perform unrelated tasks, others found themselves gaining new roles. For example, one social worker was asked to help physically transfer patients from bed to commode and noted it was “just not something that I would ever envision myself needing to help with.” The most commonly reported responsibility was being tasked with screening patients, visitors, and staff for COVID-19 symptoms when entering their building or facility. The screenings ranged from temperature checks and symptom questionnaires to disseminating protective equipment (i.e., masks and hand sanitizer). Others described having to clean more or perform technical tasks. For example, a long-term care social worker noted being asked to respond to residents’ in-room needs, such as resetting a television or reconnecting a telephone. This social worker labeled these tasks as “odd” but noted that she was motivated to help because they were isolated. Some social workers believed the lack of understanding of their roles from other Interdisciplinary team members resulted in being asked to perform outside their scope. For instance, a hospital social worker identified clinical interventions as her primary role but stated, “Doctors, they don’t understand I that we are licensed to do that”. Collectively, participants performing tasks outside of their scope recognized that “a lot fell on the social workers” because of how busy other team members had become with their existing tasks. Still, some participants noted they were not asked to perform outside their scope or did not feel performing outside of their scope was unreasonable. As pointed out by one participant, “I don’t think I’ve been asked to do anything outside of social work practice. It is social work practice. It is just the nature of where this practice takes place is, you know, highly, I guess, unsafe environment at this point. But I don’t think it’s out of the realm of social work.”

***Collaboration Challenges.*** Consistently, participants stated that nurses, doctors, and other professionals were lauded as essential workers but were often overlooked. This tension posed a challenge to effective teamwork. Still, social workers viewed themselves as essential. In fact, one social worker was happy to receive an essential worker letter from her employer and thought, “Well, goodness, someone finally got it, okay, after all this time.” That social worker said that the team of nurses and doctors understood that she did more than “fill out the Medicaid form and get [rides] back and forth to an appointment.” One social worker stated, “Certainly, the nurses and physicians get all of the acknowledgment, but I think everybody [here] has a part that’s important.” This sentiment was recurring and expressed how the nonmedical community views social workers as well. Not being regarded as essential made social workers feel devalued. One stated that it gives her an “internal feeling…of being minimalized, or ignored, or even just overlooked altogether. I don’t think most people recognize that we are there and what we do and that we are essential and … are required to be there as essential workers. It infuriates me, honestly.” One participant stated, “And I was like, they keep saying the nurses and the doctors and nurses and the doctors…well, what about the social workers?” This participant described how the term “essential” created differences in pay. “But some of the nurses got different pay because they were deemed essential on a COVID floor. My own floor is a COVID floor, I don’t get any differential pay.”

When asked what would happen if social work was not around, responses included “patients would get lost in the shuffle,” and things would “get worse” if other treatment team members were asked to address psychosocial factors outside their practice scopes. For instance, one participant described an instance where a nurse attempted to handle a patient’s emotional outbursts with “harmful vernacular” but did not have the time to “sit and de-escalate.” Participants proudly described how they can see beyond the physical aspects of a chronic condition to the “emotional toll” and “mental health aspects” of it.

### 4.5. Scope of Practice: Cultural Competence

HSWs must acquire a standard of cultural competence by recognizing institutional inequities and societal-level forms of oppression. HSWs reflected on how the co-occurrence of the Black Lives Matter movement raised their awareness of race-related health disparities.

***Institutional*.** Consistently, HSWs expressed frustration with the value placed on other disciplines’ roles (e.g., nurses and doctors) above their own. Value was evidenced by inequities in benefits and pay despite that, during the pandemic, “the RNs and the social workers…do the exact same role.” This participant gave the example of social workers administrating intravenous antibiotics, too. Further, there were differences in work-from-home privileges. One HSW stated that her “direct manager worked from home citing childcare issues, but if I had childcare issues, I wouldn’t be allowed to work from home…they should have been on the lines with us.”

***Societal*.** The co-occurrence of COVID-19 and the Black Lives Matter movement drove discourse on health equity and race disparities in healthcare [31]. As one participant noted, “the Black Lives Matter movement and the fact that…there are a lot of racial disparities” resulted in some HSWs increasing advocacy efforts about these intersecting issues. For instance, a social work supervisor said about her Black colleagues that she could not “hide away from it” and chose to “acknowledge the elephant in the room” by openly addressing racism and health in supervisory meetings. Another social worker was encouraged by a growing discussion on racism and health, noting that staff in leadership positions were “taking notice now” and were asking, “What can we do about this?”

## 5. Discussion

The purpose of this study was to describe HSWs’ scope of practice during the COVID-19 pandemic. Their scope was assessed based on healthcare social work practice standards (NASW). Four practice standards and eight themes emerged from the data: knowledge and skills (care planning and intervention and social worker–patient relationship), workload sustainability (workload expansion and workload facilitators), interdisciplinary collaboration (collaborating beyond the scope of responsibilities and collaboration challenges), and cultural competency (institutional and societal). Elucidating their roles was an important endeavor because the pandemic magnified patient needs, and social work practitioners are trained to evaluate the psychosocial needs of individuals living with chronic disease and implement evidence-based treatment modalities accordingly [12].

In this study, knowledge of theories and corresponding practice skills were paramount despite the challenges posed by the pandemic. Knowledge and skills help social workers navigate an already complex system, and the pandemic exacerbated its complexities. This finding is consistent with other studies that highlighted workers’ sense of responsibility for quality care, even with the risk of contracting COVID [32]. Some challenges already noted in the literature include psychological distress [33], workplace violence [34], and insufficient equipment [35]. Still, participants in this study were committed to navigating these challenges.

As found in other studies [36,37], workload sustainability was challenging among HSWs. Heavy workloads are associated with burnout and psychological distress [38]; therefore, it is unfortunate that HSWs were asked to perform duties beyond their typical roles. Given the unprecedented nature of the pandemic and the lack of preparedness [39], it is not surprising that social workers were most commonly tasked with COVID-19 symptom screenings. Indeed, social workers risk losing their professional license for practicing outside of their scope. It is anticipated that the conversation about whether or not these tasks were unreasonable will continue and possibly result in practice protocols. For example, the Alberta College of Social Workers issued standards for social workers participating in COVID-19 medical swabbing [40]. Perhaps the primary issue is the need for training to perform such tasks properly. For instance, one participant noted confusion over the protocol, and patients with temperatures greater than 100 were allowed to enter. Meanwhile, another pointed to a clear protocol: patients with fevers were immediately escalated to the nursing staff for further evaluation.

Interdisciplinary collaboration was necessary to navigate through the pandemic. Previous research found a lack of understanding of social work’s role and scope, a lack of respect from colleagues, differences in compensation, and working conditions prevent successful interdisciplinary teamwork [8,9,41,42]. This study’s findings revealed the same. Therefore, the following two recommendations may improve social workers’ experiences in hospitals, dialysis centers, long-term care facilities, and other healthcare settings. First, as social workers redefine the boundaries and thresholds of where social work starts and ends, physicians, nurses, and other team members should expect to respect those boundaries by better understanding the social work role. Second, like other professionals with clear guidelines and language, social workers welcome allies to advocate for more precise language in Medicare Conditions for Coverage.

Cultural competence is a long-time value in social work [43] and healthcare [44]. Typically, cultural competence is viewed as a micro-level skill. However, this study revealed that HSWs are thinking across all systems. Specifically, participants expressed the need for more dialogue and attention to what has been described as the “dual pandemic” of racial injustice and COVID-19 [45]. HSWs are well poised to lead in these efforts, given their training on social justice, systems of oppression, and, recently, anti-racism.

### 5.1. Societal Implications

Integrating social work into healthcare has become a societal priority [46]. Healthcare is equipped to respond to individuals’ health needs but inadequately prepared to address SDOH contributing to health outcomes [47]. Unfortunately, ambiguity and skepticism about social work’s values, historical background, educational training, and role in healthcare will delay the uptake of social care into the provision of medical services. Furthermore, it is unfortunate that a lack of understanding of the importance of social work before the pandemic contributed to HSWs practicing outside of scope and facing challenges in interdisciplinary care. This study highlighted those challenges through the perspectives of 54 HSWs. A follow-up study could determine if these trends persisted beyond 2020.

### 5.2. Limitations

There are three limitations to this study. First, although generalizability and causal claims are not the goals of qualitative research, this study’s findings represent participants’ contexts only. Inference is cautioned because the sample lacked geographic variability, likely because the survey was initiated in the southeastern United States. Second, the co-PIs attempted to recruit broadly, but most participants were in the U.S. South, where both co-PIs reside. Nonetheless, this dataset comprises 54 participants representing hospitals, outpatient facilities, and other healthcare settings. Third, this study did not control for covariates that may have contributed to variation in social workers’ experiences (e.g., caseload and organizational structure). Still, data were obtained on social workers’ settings, licensure, geographic location, and degree to present some nuance.

## 6. Conclusions

In this study, HSWs described how their practice scopes were stretched or refined during the pandemic. Some viewed this reality as an “all hands on deck” approach, while others experienced frustration by practice expectations. The findings add a deeper understanding of the roles social workers perform, how social workers think about these roles, and how social workers want to be understood.

HSWs are poised to address social and environmental conditions contributing to health outcomes. COVID-19 intensified these factors and further marginalized communities where poverty, poor housing, food insecurity, and other SDOH were widespread [48]. The findings in this study suggest that HSWs are best utilized in ways consistent with their training and expertise. Moving forward, healthcare systems may consider well-delineated roles and responsibilities for everyday practice and during pandemics.

## Figures and Tables

**Table 1:** Health social workers’ demographics.

Characteristic	N	%
Gender		
Male	4	7.4
Female	50	92.6
Race or Ethnicity		
Black	15	27.8
Asian	1	1.9
White	32	59.2
Hispanic	6	11.1
Education *		
BSW	1	1.9
MSW	53	98.1
License **		
LMSW	23	42.6
LCSW	23	42.6
Unlicensed	8	14.8
Age, in Years		
23–30	26	48.1
31–39	16	29.6
40–58	12	22.3
Setting		
Dialysis/Transplant Center	5	9.3
Hospital	35	63.0
Other Outpatient Setting Long-Term Care	8	24.1
Hospice	2	3.6
Region		
Midwest	4	7.4
North	11	22.2
South	31	57.4
West	7	13.0

* BSW = Bachelor of Social Work, MSW = Master of Social Work. ** LMSW = Licensed Master Social Worker, LCSW—Licensed Clinical Social Worker.

**Table 2:** Social work practice standards.

Scope of Practice Area	Theme
Knowledge and Skills	Care Planning and Intervention
	Social Worker-Patient Relationship
Workload Sustainability	Workload Expansion
	Workload Facilitators
Interdisciplinary Collaboration	Collaborating Beyond Scope of Responsibilities
	Collaboratory Challenges
Cultural Competence	Institutional
	Societal

## Data Availability

Data available on request due to privacy and ethical restrictions.

## Appendix A. Interview Questions

1. This is a study about medical social work. What is medical social work practice?
  - What are medical social workers responsible for?
2. Describe the scope of your work in your healthcare setting. You may start with talking about your setting and then your role in that setting.
3. Describe your typical day before the COVID pandemic began.
4. What aspects of your job have changed since COVID?
  - Are you serving the same type of clients?
  - Have your hours changed?
  - Are you working from home or on-site?
5. What things have you been asked to do that are outside of your scope of work, if any?
  - Do you have resentment about not having the opportunity to work from home?
6. What is it like working with the interdisciplinary team during COVID?
  - How are you conducting interdisciplinary team meetings now?
  - How is this different from before COVID?
7. What does “essential worker” mean to you?
  - What would happen if social work wasn’t there?
  - What is the risk of having to be there?
  - Do you feel valued?
  - Are you receiving hazard pay?
8. Other social workers have discussed the stress and frustration associated with working during COVID. What has been your reaction?
  - How do you balance personal life and work life?
  - What are your self-care practices?
9. What is most important about your social work role during the pandemic?
  - What type of ethical dilemmas do you encounter in your work?
  - Are those the same or different from COVID?
10. The social work code of ethics calls us to achieve social justice. What injustices have you observed during COVID?
11. How should you have been prepared in your healthcare setting?
12. How should you have been prepared in your academic curriculum?
13. Imagine you were talking to your social work professors. What would you tell them about how they should train social workers in the classroom to prepare for a pandemic?
14. As an individual, what personal characteristics, values, or skills are helpful to have as a medical social worker?
15. Is there anything else you would like to share about your experience as a healthcare professional during COVID?
16. What was your motivation for participating in this study?
